# A genome-wide structure-based survey of nucleotide binding proteins in *M. tuberculosis*

**DOI:** 10.1038/s41598-017-12471-8

**Published:** 2017-10-02

**Authors:** Raghu Bhagavat, Heung-Bok Kim, Chang-Yub Kim, Thomas C. Terwilliger, Dolly Mehta, Narayanaswamy Srinivasan, Nagasuma Chandra

**Affiliations:** 1 Department of Biochemistry, National Mathematics Initiative, Bangalore, India; 2 Molecular Biophysics Unit Indian Institute of Science, Bangalore, 560012 India; 3 Bioscience Division, Los Alamos National Laboratory, Los Alamos, New Mexico, 87545 USA

**Keywords:** Computational biology and bioinformatics, Computational biology and bioinformatics, Diseases, Diseases

## Abstract

Nucleoside tri-phosphates (NTP) form an important class of small molecule ligands that participate in, and are essential to a large number of biological processes. Here, we seek to identify the NTP binding proteome (NTPome) in *M. tuberculosis (M.tb*), a deadly pathogen. Identifying the NTPome is useful not only for gaining functional insights of the individual proteins but also for identifying useful drug targets. From an earlier study, we had structural models of *M.tb* at a proteome scale from which a set of 13,858 small molecule binding pockets were identified. We use a set of NTP binding sub-structural motifs derived from a previous study and scan the *M.tb* pocketome, and find that 1,768 proteins or 43% of the proteome can theoretically bind NTP ligands. Using an experimental proteomics approach involving dye-ligand affinity chromatography, we confirm NTP binding to 47 different proteins, of which 4 are hypothetical proteins. Our analysis also provides the precise list of binding site residues in each case, and the probable ligand binding pose. As the list includes a number of known and potential drug targets, the identification of NTP binding can directly facilitate structure-based drug design of these targets.

## Introduction

Nucleoside tri-phosphates (NTP) participate in many cellular processes, regulate signal transduction events, mediate a number of transport reactions and play a role in maintaining the cell structure^1–3^. They form a vital part of many metabolic reactions and regulate many other processes like nucleotide synthesis and cell signaling^1,4^. Adenosine triphosphate (ATP) is also ubiquitously conserved as the currency molecule in biological cells and used as a phosphate donor for phosphorylation of various proteins^1,2,4^. Besides these, ATP is also known to serve as an allosteric modulator of a number of proteins^5,6^. Several ATP binding proteins are known to be essential in different organisms and have been explored for their potential as drug targets of antibacterial and anticancer agents^7^. Although a number of proteins that bind to ATP are known in many organisms, the entire repertoire of ATP binding proteins (ATPome) in a given cell, remains incompletely characterized. A systematic profiling of the whole proteome to find the ATPome is of great interest, and will impact drug target identification. Identification of the ATPome will also lead to a more complete annotation of many hypothetical and otherwise uncharacterized proteins that may have been associated with the disease in some form as identified by gene expression or genome-wide association or related studies, thereby providing a larger pool of proteins as a base for target selection. Considering the whole ATPome will also be useful in identifying drug targets that can achieve better selectivity. Chemical proteomics approaches have been explored in some organisms in which a chemical probe is used to capture ATP binding proteins, which are subsequently characterized through digestion and mass profiling^8–10^. These methods have provided a high-throughput means of identifying ATP binding proteins. However, the methods have their limitations such as many challenges in probe design, capture coverage, capture strength and detection sensitivity, which remain to be addressed.

*Mycobacterium tuberculosis (M.tb)*, the causative for tuberculosis is a deadly human pathogen that leads to more than 2 million deaths every year^11^ (Global tuberculosis report 2016). Although a handful of drugs are available and are used widely in the clinic, the emergence of drug resistance is posing a major problem in the management of the disease, warranting discovery of new drugs^12,13^. Towards this, identification of new drug targets in the pathogen is of great interest, which is being explored from multiple angles^14–16^. The genome sequence of this bacterium was one of the earliest whole genome sequences to be determined^17^. Although there has been significant progress in genome annotation since then, a number of proteins remain unannotated or incompletely characterized. This is particularly true for many hypothetical proteins. High confidence annotations are key for identifying novel drug targets and informing new drug discovery efforts. Given the importance of ATP, several ATP binding proteins are expected to be viable drug targets. Bedaquiline, a drug that has recently entered the market, inhibits ATP synthase in mycobacteria^18^. Other proteins such as icl, pcaA and ddlA are being explored as drug targets^19–22^. Other nucleotide binding sites, are also likely to be important as drug targets, but are much less explored^23^. A challenge in targeting NTP binding proteins however, is to find those proteins that are sufficiently different from the host proteins, so as to enable design of selective inhibitors. A genome-wide identification of the NTPome in *M.tb* will be very useful that can be used to search for possible targets which have a high potential for achieving selectivity.

A chemical proteomics approach has been recently reported that utilizes a desthiobiotin-conjugated ATP as a molecular probe that captures target enzymes that are previously covalently modified with biotin in their nucleotide binding domains^24^. The captured proteins are subsequently digested with trypsin and labeled peptides are enriched via streptavidin affinity capture beads and subjected to LC-MS/MS for the identification of ATP-labeled proteins^24^, and 539 proteins are identified through this approach. Another method that has been used for the same purpose involves the use of an activity-based probe to annotate and validate ATP binding proteins. About 317 ATP binding proteins are identified from this method^8^. A quick comparison of the list of proteins produced by the two methods indicates that only a small fraction is common to both, clearly suggesting that a number of false negatives exist in both the methods. New orthogonal methods are necessary for independent and systematic identification of the ATP binding proteins and also for other nucleotide binding proteins. In this work, we explore two independent approaches, (a) a computational screen that identifies proteins that have NTP binding structural signatures and (b) a chemical proteomics screen that identifies NTP binding proteins in *M.tb* cell extract.

Bioinformatics methods which can screen the proteomes from multiple perspectives offer a great benefit in terms of being comprehensive, the ease of screening, the speed and control over the sensitivity of detection. Use of protein structures, chemical similarity and network-based dynamics are being increasingly used in protein function annotation, drug development process and understanding the molecular bases of disease^25–27^. A commonly used computational probe is a sequence motif, an example being the P-loop containing Walker motif that is characteristic of ATP binding^28,29^. However, it is clear that the presence of such a sequence motif is neither a necessity nor a sufficient criterion for ATP binding. Many proteins that do not contain this motif are known to bind ATP. A search for the Walker motif in the genome sequence of *M.tb* fetches only 161 proteins. Structural motifs are typically far more superior to sequence motifs as they are more conserved and more reflective of a given binding function than the sequence motifs. This is especially true for ATP binding sites as a diverse array of proteins bind to ATP. However, there are many hurdles to cross in order to utilize structural motifs, which are (a) structural data of proteins is required at a proteome-scale, which is far from trivial b) the structural motifs need to be defined clearly c) structural motifs must be specific towards a given ligand recognition, and (d) sensitive methods are required to compare structural motifs against protein structures at a proteome-scale. In our laboratory, we are equipped with resources and methods for all four aspects. We have previously modelled protein structures in *M.tb* for about 70% of the proteome^25^ and have developed a suite of algorithms to detect binding pockets^30^, compare and match the binding pockets^31,32^, superpose binding sites^33^ and combine them into a workflow to obtain structure-based function annotations^34^. Recently, we have carried out a large-scale analysis of 4,766 ATP and other NTP binding proteins from PDB and have grouped all known NTP binding sites into 27 different site-types, and have derived a structural motif or a site-signature for each type^35^. The known NTP binding proteins from the PDB comprise of members from a minimum of 374 sequence families and 145 structural folds. Such a large diversity in these makes it difficult to use sequence-based or fold-based methods for identifying NTP binding proteins. On the other hand, the three dimensional structural motifs at the binding sites that we have derived are very useful for this purpose. In the laboratory, we have previously used such an approach to obtain a structural annotation of the *M.tb* proteome^25^, identify characteristic features of sialic acid binding proteins^36^ and also for other sugar binding proteins^37,38^.

In this work, we use the 27 site signatures as search keys and carry out a genome-wide survey, to the extent covered by the structural models. From this, we obtain a set of 1,768 proteins that can be identified as NTP binding proteins. Some of these are identified by the chemical proteomics approaches as well. In addition, we find many more, several of which have indirect evidence from literature, indicating that we have much improved sensitivity as compared to those methods. We then apply a biochemical screen, based on dye-ligand affinity chromatography (DLAC), and experimentally validate 47 more NTP binding proteins. We thus present a combined computational proteomics and a biochemical approach that identifies ATP binding proteins in any proteome, where structural models are provided as an input. In addition, our computational approach also identifies guanosine triphosphate (GTP), cytidine triphosphate (CTP), uridine triphosphate (UTP) and thymidine triphosphate (TTP) binding sites in the proteome. Our analysis leads to a rich comprehensive resource of nucleotide binding proteins in *M.tb*. Since the identification is made on the basis of binding site structures, the binding site location and the individual amino acid residues are also explicitly identified in each protein.

## Results

### Binding sites comparison and identification of the ‘NTPome’

The structural models of 2877 proteins amounting to 70% of the *M.tb* proteome, which were generated by us previously, contained a total of 13858 small molecule binding pockets. The pockets identified were of high confidence as they were detected as consensus by three independent binding site prediction methods based on energetic considerations, geometric parameters and evolutionary information (See Methods: section 4.1). Screening the *M.tb* pocketome using each of the 27 structural motifs yielded 1,768 hits, which we refer to as the ‘NTPome’ hereafter. Supplementary Table ST1 lists all these proteins, along with their functional categories. The number of hits is indeed dependent on the selected threshold for considering a pair of sites as similar. A higher threshold will identify fewer but more confident hits but will have a large number of false negatives whereas, a lower threshold will lead to more hits but with a number of false positives. The selected threshold is expected to be a trade-off on the false negatives so as to minimize false positives. Supplementary Table ST2 shows the number of proteins identified with different thresholds, whereas Supplementary Table ST3 lists the highest PMS (PocketMatch Score) each of the 1,768 proteins share with the queried NTP motif. Higher the PMS, higher is the similarity and higher is the confidence.

In order to understand how well our method was performing, we tested whether the *M.tb* proteins that were complexed to one of the NTPs and whose structures are available, were correctly identified by our approach. The Protein Data Bank^39^ (PDB) contains 108 entries for *M.tb* proteins bound to NTP ligands, which correspond to crystal structures of 35 different proteins. 18 of them are bound to adenine-based nucleotide ligands, which means these 18 are adenine specific, 3 entries are specific to GDP, 2 are specific to CTP, 4 proteins are specific to uracil-based nucleotides and 2 are specific to TTP. The remaining 6 proteins are bound to more than one type of NTP ligand. For all these entries, the information of their ligands or binding site residues was removed before scanning for NTP binding sites. In other words, for these proteins, an independent prediction for NTP binding potential was carried out using only the apo-protein structure. 30 out of these 35 proteins were in fact identified as NTP binding proteins using our approach. It was also verified that the location of the pocket and the binding site residues were correctly identified in our predictions. This served as a first-step validation exercise, demonstrating that known proteins are correctly identified as hits for NTP binding. The 5 proteins that were missed fall into the category of false negatives at the given threshold, and can be identified if partial similarities are considered. In order to minimize false positives, we have restricted to whole site similarities and have not used partial similarities as criteria for identifying NTP binding proteins.

We then tested if our predictions picked up the correct ligand as well. We scanned the predicted sites for these proteins against all the known NTP binding sites from PDB at the binding site level, and the ligand from the highest scoring hit was taken as the ligand for the protein being studied. Adenine-based nucleotide was correctly identified as the ligand in 17 out of 18, GDP in 3 out of 3, TTP in 2 out of 2, and uracil-based nucleotide in 2 out of 4 by our method. It was further seen that CTP ligand was identified as hit in both the cases at a slightly lower threshold of PMSmax 0.48, for one protein, and 0.47 for another. This also meant that the motifs correctly identified the specific ligands that were actually seen in PDB, further suggesting the sensitivity of discrimination of purine and pyrimidine based ligands, and the motifs captured the subtle differences wherever possible.

The query motifs, once derived, are in essence a group of amino acids at the binding site, and are not explicitly tagged with a ligand and hence no direct information of the specific ligand is used while screening. In order to get an estimate of the most likely ligand for each of these binding sites in the NTPome, each predicted NTP site was taken and reverse screened against known NTP binding sites from PDB and the ligand of the highest scoring hit was taken as the associated ligand for the predicted site. From this, 1,286 could be associated with ATP as the topmost ligand hit, 250 with GTP, 102 with CTP, 86 with TTP and 44 with UTP.

### Distribution of protein classes in the NTPome and preference of certain motifs

**Figure 1:**
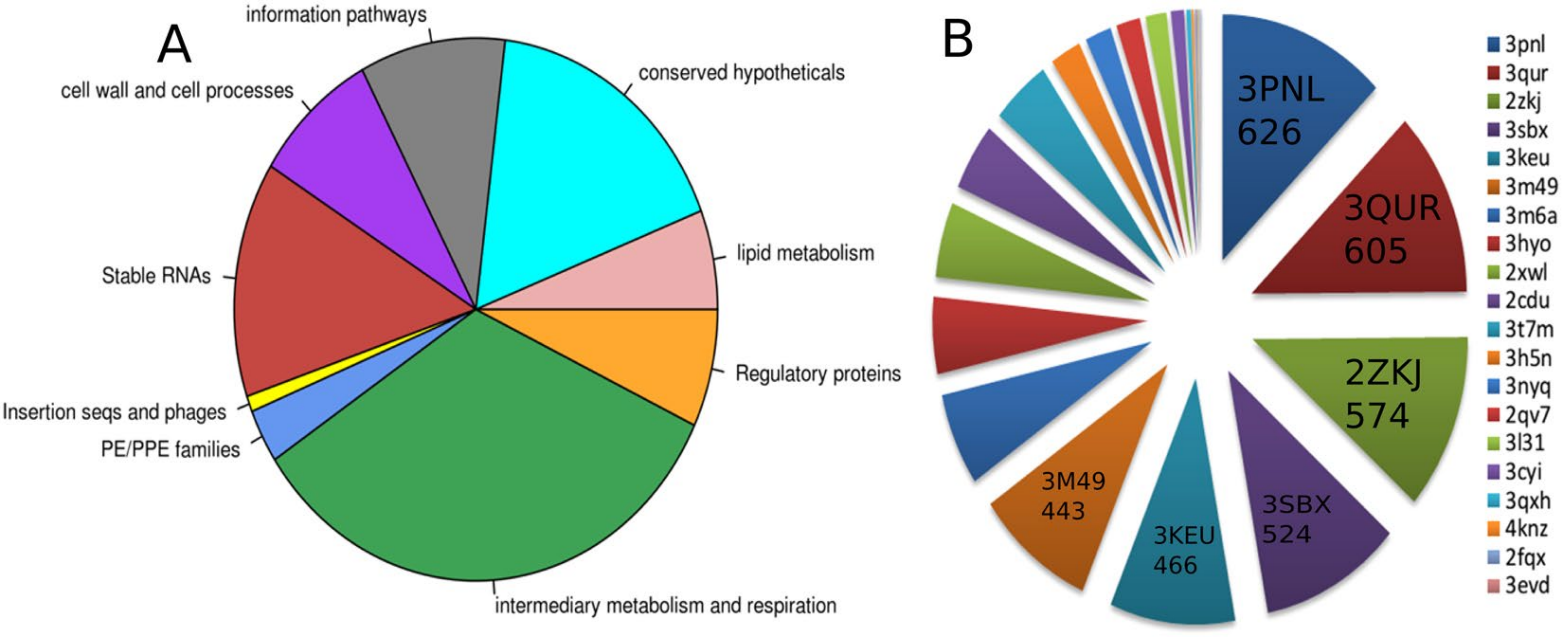
(**A**) Pie-chart showing the different tuberculist functional classes for *M.tb* proteins that were identified in the NTPome. It was indeed interesting to see a major portion of the hypotheticals that were identified, which could serve as a possible function annotation at the molecular level of NTP binding for these proteins. Same is with the PE/PPE family proteins which are not known to bind NTP. (**B**) Distribution of identified NTP motifs in the *M.tb* proteome. Although 24 motifs were seen across the different proteins in NTPome, there was a preference for certain motifs, namely 3PNL-like motif, 3QUR-like, 2ZKJ-like, 3KEU-like and 3SBX-like motifs. 3PNL, 3QUR, 2ZKJ, 3KEU and 3SBX refer to the PDB codes of the representative proteins in the study of identifying NTP motifs that was carried out previously, and the number in each sector refers to the total number of *M.tb* proteins identified for that particular motif.

In comparison to the known NTP binding sites in *M.tb*, the hits in the NTPome is a phenomenal increase in number, indicating that more than one third of the proteome harbours NTP binding sites. Not surprisingly, 610 of these are enzymes involved in the intermediary metabolism and respiration class. Several proteins related to cell wall and cell processes including transport proteins were also identified to harbour NTP binding sites. Figure 1a shows the tuberculist distribution of functional classes of the predicted NTPome. A KEGG mapper representation of the various pathways that are enriched and more represented in the NTPome is shown in Supplementary Fig. S1. From this, it can be seen that lipid metabolism, nucleotide metabolism, amino acid metabolism and carbohydrate metabolism form the major pathways that are seen in the NTPome. It was also interesting to note that many proteins belonging to the hypothetical category were identified in our NTPome. The presence of NTP binding motifs in them provides a clue about their function. Another category of proteins of interest in the *M.tb* genome is that of the PE/PPE family. 58 of these proteins were identified among the hits in our analysis, which could not be identified through sequence based searches.

Since a genome-wide scan with 27 different binding motifs was carried out, it was of interest to see how many of these 27 motifs were present in *M.tb*, and what are their relative frequencies. It was observed that 24 of the 27 motifs were identified in *M.tb*. However, a strong preference was seen for some motifs, especially for the motif represented by a) DhaL-like with DAK1/DegV-like fold (represented by site-type 3PNL) 35%, b) Carbamate kinase-like fold (represented by site-type 3QUR) 34%, c) His-Me finger endonucleases (represented by site-type 2ZKJ) 32%, and d) MCP/YpsA-like (3SBX) 30%.

The 27 site-types identified for NTP binding represented 4766 proteins belonging to about 145 different structural folds. Even though they were representatives of the entire repertoire of NTP binding space of proteins, it was seen that among the 27 types, there exists some partial similarities. Using these part-similarities, a super-classification of 27 site-types into 9 super-types was further carried out in our previous study^35^. It was further interesting to note that majority of the NTPome which exhibits significant similarities with the site-types 3PNL, 3QUR, 2ZKJ and 3SBX, belonged to the same super-type. In other words, this super-type is seen to be predominant in the NTPome. The other preferred site-types of 3M6A, 3M49, 3T7M and 2CDU that occur in NTPome belong to different super-types. However, for the purposes of identifying similarities among diverse classes and function annotation, consideration at the super-type level was not found to be very insightful as it will preclude the use of many important residues in the analysis, which is best obtained at the level of site-types. Hence for function annotation and all further analysis, the entire NTPome was analyzed at the level of individual site-types and not at the level of super-types.

**Figure 2:**
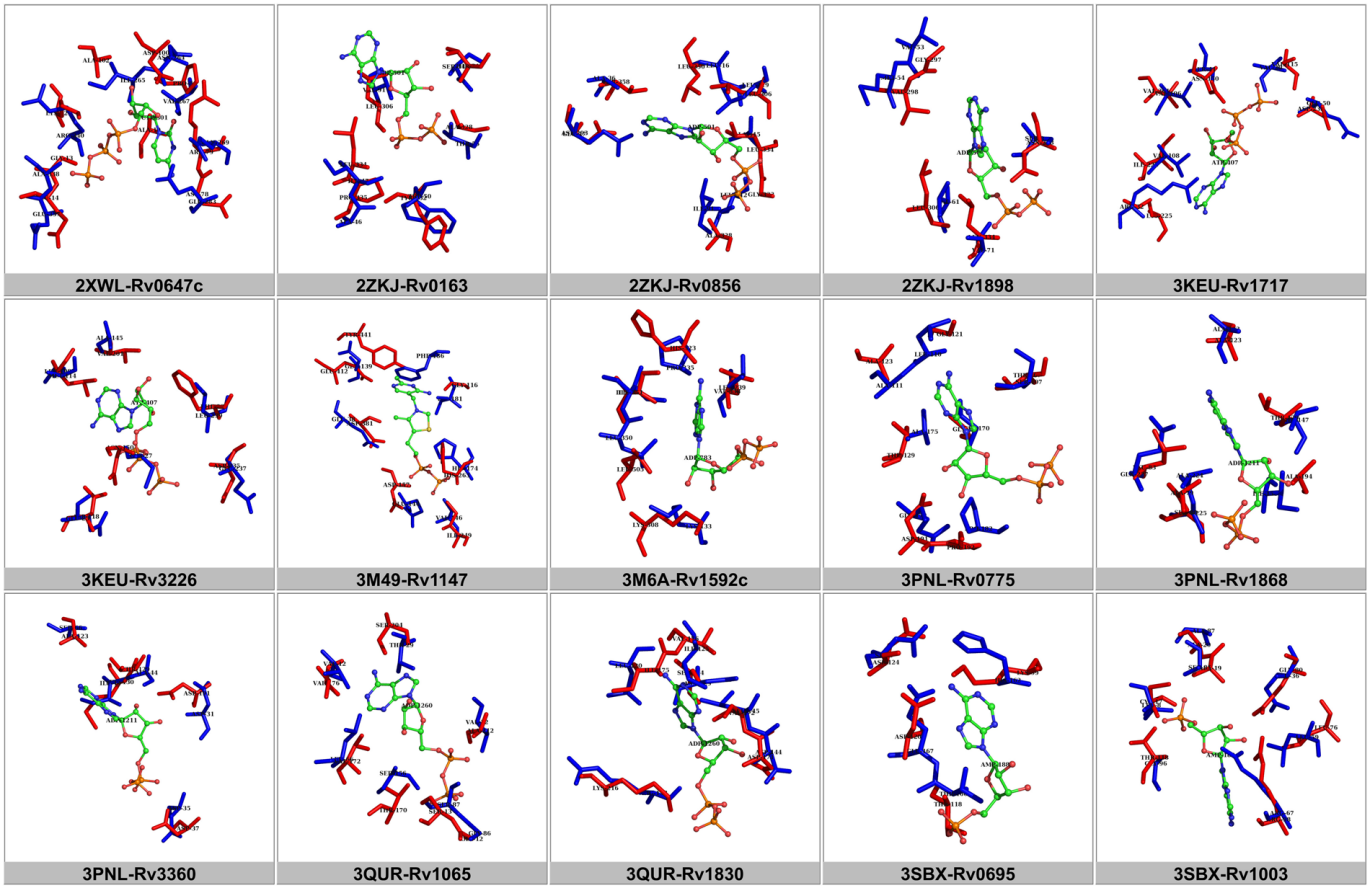
Pair-wise superpositions for 15 pairs of proteins that fall under the category of hypotheticals. The reference NTP motif is shown in red sticks and sites belonging to *M.tb* proteins are shown in blue stick representations in each panel, with the ligand shown in ball and stick model. It can be seen that there are not only identical residue matches between the sites in some cases, but also, similar geometrical orientations of the side chain of amino acid residues, further strengthening the possibility of NTP binding by these proteins. It has to be noted that the proteins in the pair do not share any relatedness in their sequences and folds.

The distribution of the 24 motifs across the NTPome is shown in Fig. 1b. Figure 2 shows the binding site superpositions for 15 examples from the hypothetical category that were identified as hits for NTP binding. Supplementary Fig. S2 shows the alignments for 8 pairs of proteins belonging to the PE/PPE family which are identified as hits.

### Experimental testing of NTP binding for selected proteins

We then used an independent experimental method to test if we can get validation for any proteins predicted by our computational method. A high-throughput method of using Cibacron Blue F3GA dye-ligand affinity chromatography (DLAC) described previously was carried out^40,41^. In this method, initially we used *M.tb* cell extract proteins to bind on the dye and elute with nucleotide ligands after intensive washing, and the proteins eluted by nucleotides were analyzed by 2D-gel analysis and mass spectroscopy (MS) for identification of each eluted protein shown on 2D-gel. To confirm the 2D and MS data, for some proteins that we could purify, we tried the DLAC process again using the purified *M.tb* proteins. According to these purified proteins data, some part of NTPome data were obtained by the DLAC solely with *M.tb* proteins purified without 2D-gel and MS process because the IDs of purified proteins are known. The ligand data obtained by DLAC was applied to improve crystal quality of *M.tb* proteins to be able to solve their structures^40^.

**Figure 3:**
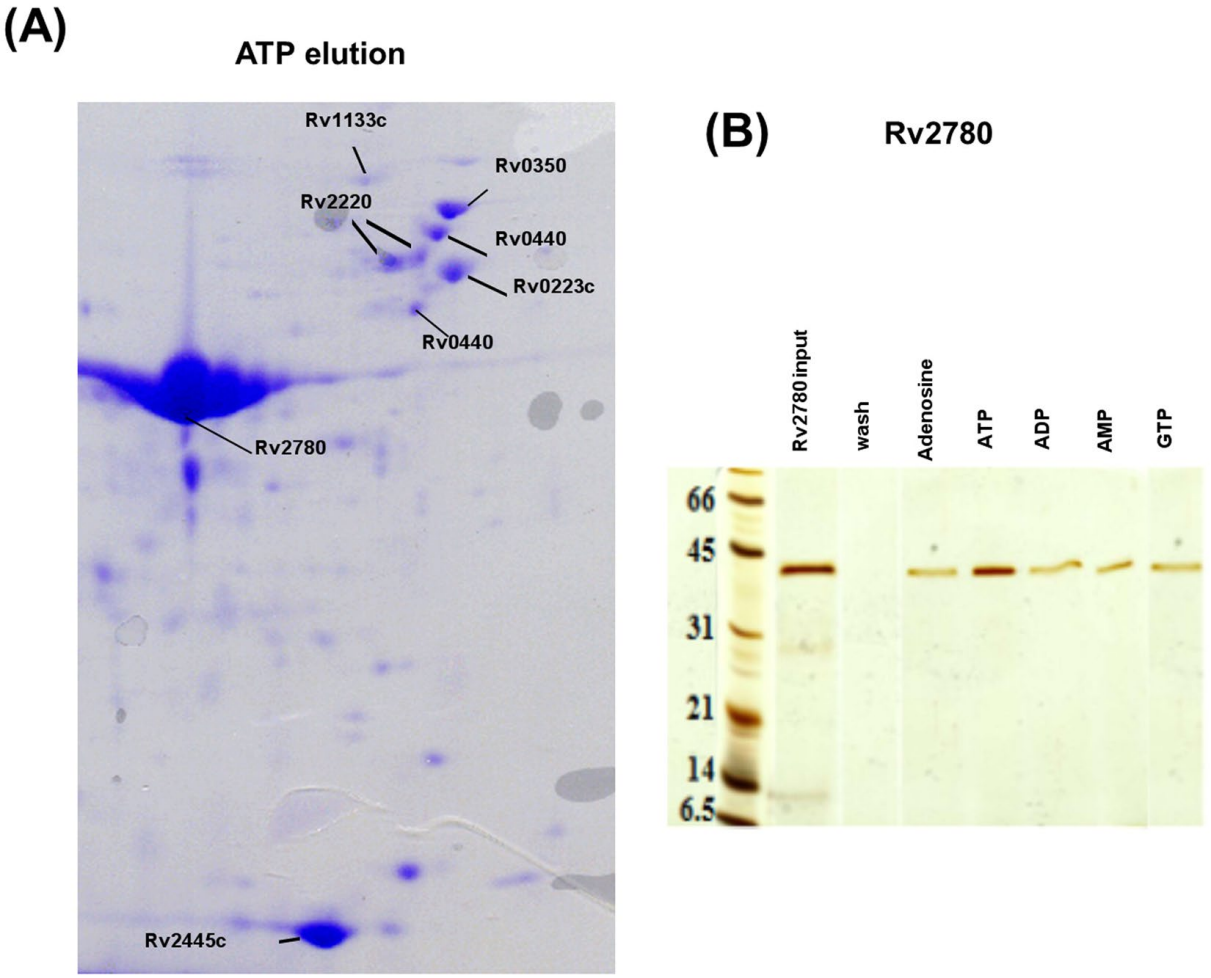
(**A**) 2D-gel image of *M.tb* cytosolic DLAC fraction eluted by ATP. The identified *M.tb* proteins are labeled on the representative protein spots with their Rv IDs. The Rv IDs of the rest of spots are listed in Table 1. This image shows part of original 2-D gel in the range of MW 100-10 kDa and pI 6–4.5, which covers most of the DLAC eluted proteins by ATP. (**B**) 1D SDS-PAGE of NTP ligands interaction analysis by DLAC using purified Rv2780. Each lane shows the fraction obtained during the process of DLAC including recombinant Rv2780 loaded on to Cibacron Blue F3GA dye resin (Rv2780 input), the fraction after washing with column buffer (wash) and Rv2780 protein eluted by five NTP ligands (Adenosine, ATP, ADP, AMP and GTP) showing the molecular weight marker on left. The Rv2780 band shown in each ligand lane indicates the interaction of applied ligand. From the darkness of bands –protein amount eluted by each ligand, the degree of each ligand’s interaction with Rv2780 can be also obtained.

**Figure 4:**
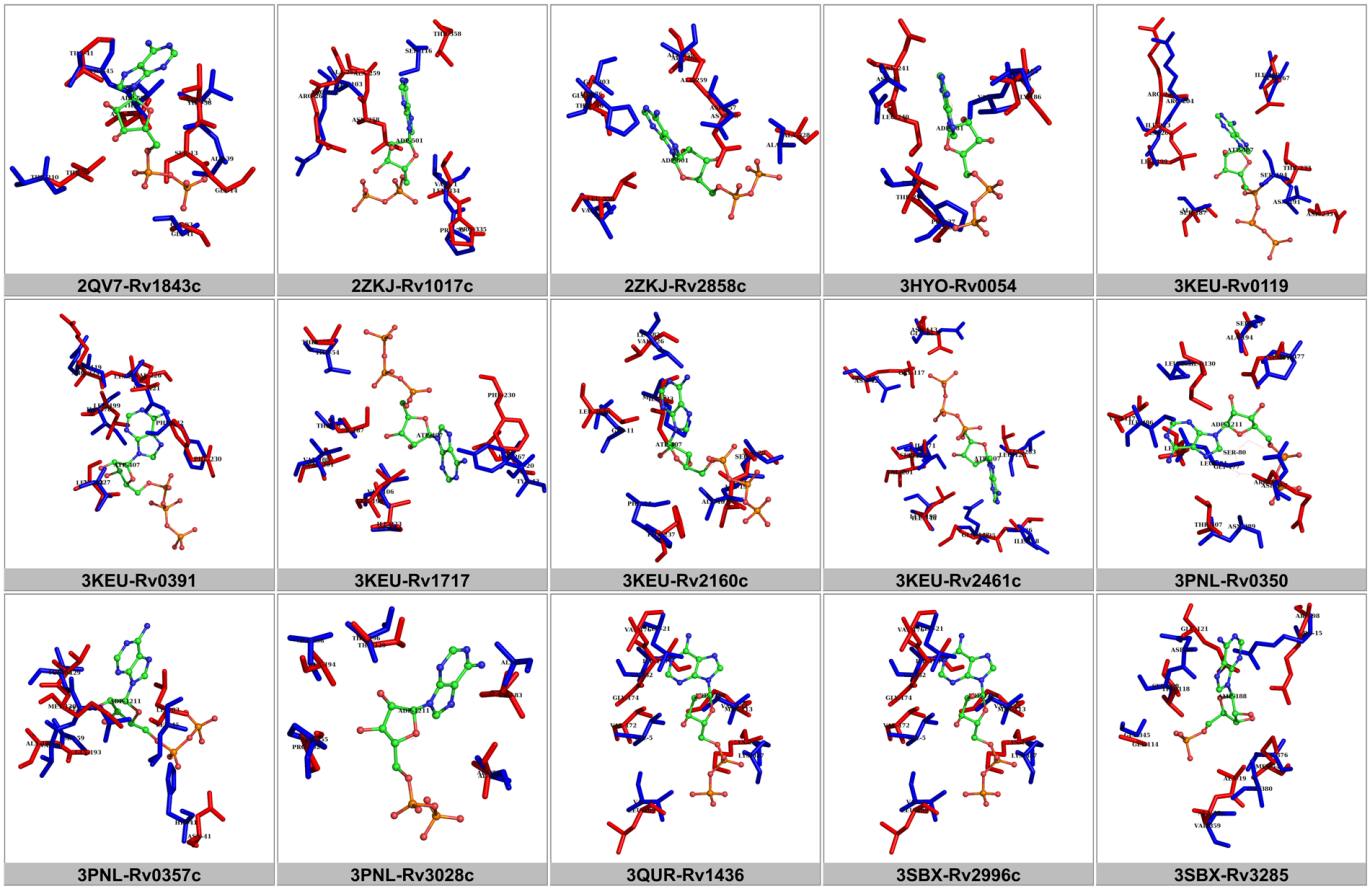
Pair-wise alignments of 15 pairs of proteins that were successfully tested for NTP binding using DLAC technique are shown. It has to be noted that the two proteins in a pair are not closely related by their sequences, but share a significant similarity at their binding sites. In all panels, the reference NTP motif is shown as red sticks and the aligned pocket residues of *M.tb* protein are shown in blue sticks with the ligand shown in ball and stick representation colored by atom type. With this information, we can also get more details on the possible mode of NTP binding in these proteins along with the exact locations on the protein surface defined by a set of pocket residues.

It has to be noted that the chemical proteomics approach that has been adopted using DLAC technique is a high-throughput method that utilizes cytosolic extract or membrane fraction. We have also reported that only 40% of cytosolic protein bound to the resin and could be detected, as determined by Bradford assay. Hence, it is difficult to answer the question of how many proteins of the 1,768 in NTPome were actually tested for NTP binding. Using the DLAC approach, we have successfully tested NTP binding for 47 proteins from the predicted list, which is still a very encouraging number. As examples of NTP interaction analyses by DLAC, Fig. 3A shows 2D gel of *M.tb* cytosolic extract proteins eluted by ATP, and in Fig. 3B, the result of DLAC performed with purified Rv2780 is shown in 1D gel and confirmed its interaction with ATP as well as other ligands like adenosine, ADP, AMP and GTP. Table 1 lists the set of proteins that show interaction with the NTP ligands as tested by DLAC. Figure 4 shows binding site alignments of 15 pairs of proteins with their respective query motifs, that have been experimentally validated using the experimental approach. It is very convincing to see that of the 47 proteins tested successfully for NTP binding, 4 of them are hypotheticals, and are reported for NTP binding for the first time by us.

**Table 1:** showing the list of *M.tb* proteins that were verified for NTP binding using DLAC. The different NTP ligands that showed binding for each protein are mentioned in the last column. The four proteins belonging to the hypothetical category are shown in bold-face.

Rv ID	Protein Name	NTP ligands identified
Rv0054	Single-strand binding protein Ssb (helix-destabilizing protein)	GTP
Rv0119	Probable fatty-acid-CoA ligase FadD7 (fatty-acid-CoA synthetase) (fatty-acid-CoA synthase)	AMP ADP GTP
Rv0350	Probable chaperone protein DnaK (heat shock protein 70) (heat shock 70 kDa protein) (HSP70)	AMP ATP GTP
Rv0357c	Probable adenylosuccinate synthetase PurA (imp-aspartate ligase) (ADSS) (ampsase)	ADP ATP GTP
Rv0391	Probable O-succinylhomoserine sulfhydrylase MetZ (OSH sulfhydrylase)	GTP
Rv0440	60 kDa chaperonin 2 GroEL2 (protein CPN60-2) (GroEL protein 2) (65 kDa antigen) (heat shock protein 65) (cell wall protein A) (antigen A)	ATP
Rv0467	Isocitrate lyase Icl (isocitrase) (isocitratase)	AMP ADP GTP
**Rv0500A**	**Unknown function**	**AMP GTP**
Rv0672	Probable acyl-CoA dehydrogenase FadE8	ATP GTP
Rv0859	Possible acyl-CoA thiolase FadA	AMP ADP ATP GTP
Rv1007c	Methionyl-tRNA synthetase MetS (MetRS) (methionine-tRNA ligase)	AMP ATP GTP
Rv1017c	Probable ribose-phosphate pyrophosphokinase PrsA (phosphoribosyl pyrophosphate synthetase) (PRPP synthetase)	AMP ADP ATP GTP
Rv1023	Probable enolase Eno	ATP
Rv1133c	Probable 5-methyltetrahydropteroyltriglutamate–homocysteine methyltransferase MetE (methionine synthase)	ATP GTP
Rv1327c	Probable glucanase GlgE	GTP
Rv1383	Probable carbamoyl-phosphate synthase small chain CarA (carbamoyl-phosphate synthetase glutamine chain)	ADP ATP
Rv1391	Probable DNA/pantothenate metabolism flavoprotein homolog Dfp	ATP GTP
Rv1436	Probable glyceraldehyde 3-phosphate dehydrogenase Gap (GAPDH)	AMP ADP ATP GTP
Rv1559	Probable threonine dehydratase IlvA	ADP ATP GTP
Rv1688	Possible 3-methyladenine DNA glycosylase Mpg	ADP ATP GTP
**Rv1717**	**Conserved hypothetical protein**	**ADP ATP**
Rv1843c	Probable inosine-5′-monophosphate dehydrogenase GuaB1(imp dehydrogenase) (IMPDH) (IMPD)	AMP ADP ATP GMP GTP
Rv1908c	Catalase-peroxidase-peroxynitritase T KatG	AMP ADP GTP
Rv2029c	6-phosphofructokinase PfkB (phosphohexokinase) (phosphofructokinase)	ADP GTP
Rv2031c	Heat shock protein HspX (alpha-crystallin homolog) (14 kDa antigen) (HSP16.3)	AMP ADP ATP GTP
Rv2145c	Diviva family protein Wag31	AMP ADP ATP GTP
**Rv2160c**	**Conserved hypothetical protein**	**ADP ATP GTP**
Rv2215	DlaT	GTP
Rv2461c	Probable ATP-dependent CLP protease proteolytic subunit 1 ClpP1 (endopeptidase CLP)	ATP GTP
Rv2605c	Probable acyl-CoA thioesterase II TesB2 (TEII)	GTP
Rv2688c	Antibiotic-transport ATP-binding protein ABC transporter	ATP GTP
Rv2780	Secreted L-alanine dehydrogenase Ald (40 kDa antigen) (TB43)	AMP ADP ATP GTP
Rv2783c	Bifunctional protein polyribonucleotide nucleotidyltransferase GpsI: guanosine pentaphosphate synthetase + polyribonucleotide nucleotidyltransferase (polynucleotide phosphorylase) (pnpase)	AMP ADP ATP GTP
Rv2855	NADPH-dependent mycothiol reductase Mtr	ATP
Rv2858c	Probable aldehyde dehydrogenase AldC	AMP ADP ATP GTP
Rv2996c	Probable D-3-phosphoglycerate dehydrogenase SerA1 (PGDH)	ADP
Rv3028c	Probable electron transfer flavoprotein (alpha-subunit) FixB (alpha-ETF) (electron transfer flavoprotein large subunit) (ETFLS)	GTP
**Rv3075c**	**Conserved protein**	**ATP GTP**
Rv3273	Probable transmembrane carbonic anhydrase (carbonate dehydratase) (carbonic dehydratase)	ADP ATP GTP
Rv3280	Probable propionyl-CoA carboxylase beta chain 5 AccD5 (pccase) (propanoyl-CoA:carbon dioxide ligase)	AMP
Rv3285	Probable bifunctional protein acetyl-/propionyl-coenzyme A carboxylase (alpha chain) AccA3: biotin carboxylase + biotin carboxyl carrier protein (BCCP)	AMP ADP ATP GTP
Rv3336c	Probable tryptophanyl-tRNA synthetase TrpS (tryptophan-tRNA ligase) (TRPRS) (tryptophan translase)	ATP
Rv3389c	Probable 3-hydroxyacyl-thioester dehydratase HtdY	ATP
Rv3401	Conserved protein	AMP
Rv3457c	Probable DNA-directed RNA polymerase (alpha chain) RpoA (transcriptase alpha chain) (RNA polymerase alpha subunit) (DNA-directed RNA nucleotidyltransferase)	ATP GTP

### Comparison with experimentally identified ATP binding proteins from literature

**Figure 5:**
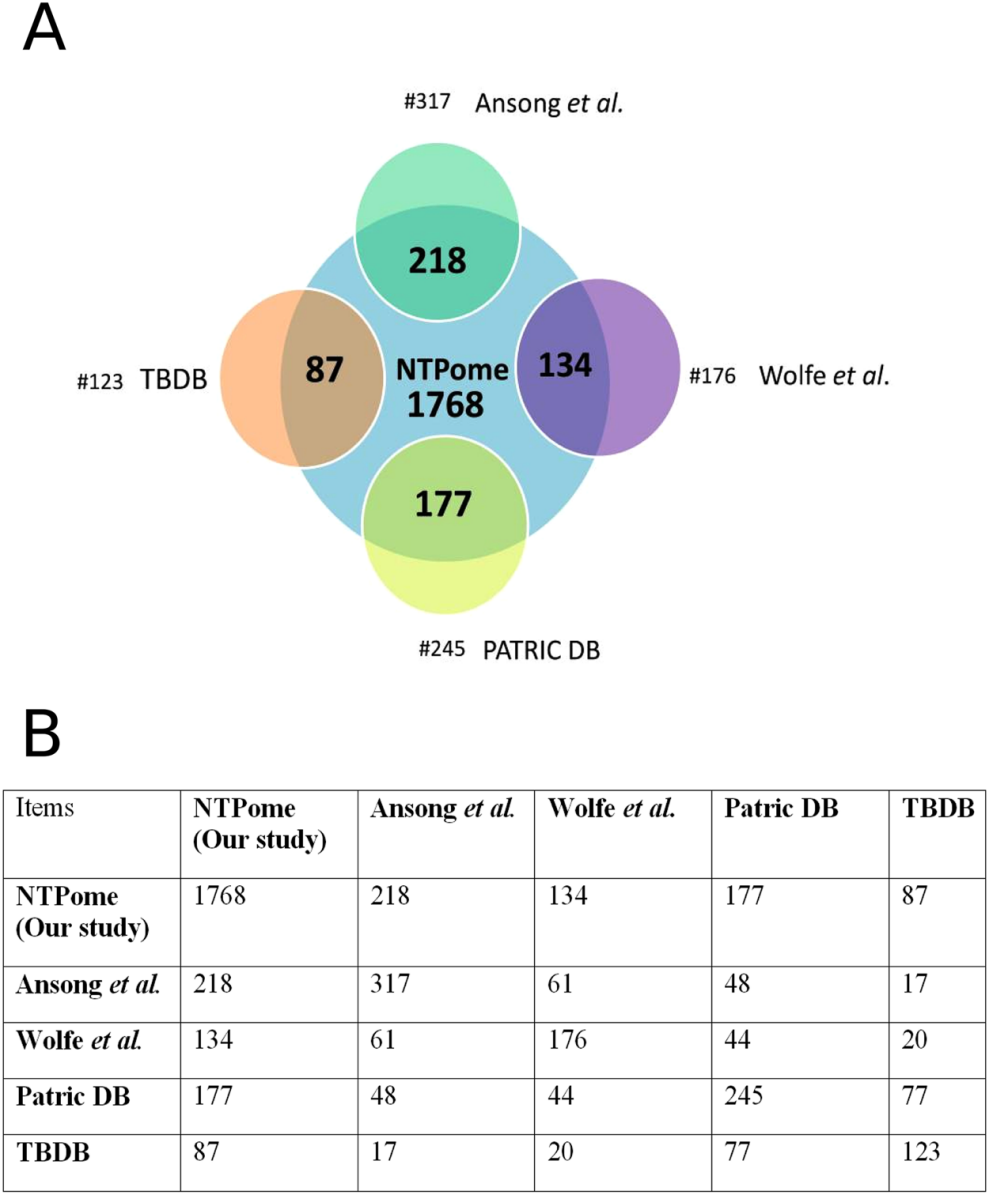
(**A**) A Venn-diagram representation showing the common number of proteins that have been identified by our study in comparison with other studies. In total, 1768 proteins were identified for NTP binding, out of which 218 are common to 317 proteins from Ansong *et al*. 134 out of 176 from Wolfe *et al*. 177 out of 245 from PATRIC DB and 87 out of 123 from TBDB. While Ansong *et al*. and Wolfe *et al*. are experimental based studies, TBDB and PATRIC databases are computational based studies. (**B**) Table showing an all-vs-all comparison of our predicted list of 1768 in the NTPome with the 4 different comparator groups. The number in each box indicates the number of common proteins identified between the pair.

We then tested if any of the predictions were supported by experimental observations from literature. Two different studies describing high-throughput identification of ATP binding proteins have been reported in literature^8,9^. A search for adenosine binding in *M.tb* by utilizing a high throughput activity-based protein profiling (ABPP), combined with sequence-based methods was reported by Ansong *et al*.^8^, where they identified 317 proteins as capable of binding to ATP. 218 of the 317 proteins were identified by our approach as well, which included 31 conserved hypothetical proteins. Wolfe *et al*.^9^ have reported a chemical proteomics approach where they use a desthiobiotin-label and carry out a shotgun proteomics analysis to identify proteins in the enriched subproteome, from which they identified around 176 proteins. Upon comparison, we found that 134 proteins are in common between our predictions and their list. By comparison with the results of these proteomics approaches with our DLAC data, we found 13 proteins identified by our method (Rv0119, Rv0440, Rv0500A, Rv1391, Rv1843c, Rv1908c, Rv2145c, Rv2215, Rv2461c, Rv2783c, Rv3028c, Rv3336c and Rv3389c), to be validated by all three proteomics methods. Two other databases namely the Patric database^42^ and TBDB^43^ are widely used and serve as excellent resources for annotations based on individual reports describing functional annotation of individual proteins. Out of the 245 proteins that were annotated for NTP binding in Patric database, 177 were correctly identified by us. Similarly, TBDB has a list of 123 proteins annotated as NTP binding, of which 87 were correctly identified by us. Figure 5 shows a summary of how our predictions fare as compared to what is known in literature. From this comparison with experimentally identified ATP binding proteins from literature, overall, the success rate of identifying a NTP binding site purely based on binding site characteristics as in this study is seen to be quite high, which is in the range of 68 to 76%.

### Function annotation for the putative NTP binding proteins currently categorized as hypotheticals and are of unknown function

The *M.tb* genome has about 1,042 proteins that are still annotated as uncharacterized, and their function still remain unknown. 294 of these were found to be in the NTPome. Four of these conserved hypotheticals, which are Rv0500A, Rv1717, Rv2160c and Rv3075c, were validated to be ATP binding proteins by us using the DLAC approach. Detecting an NTP binding pocket provides important clues about the functional roles of these proteins. In comparison to the previously reported studies described in the previous section, many new ones were identified by our approach, reflecting on the sensitivity of this method. Supplementary Table ST1 (sheet 2) lists the proteins under the category of hypotheticals and unknown function that were identified as hits for NTP binding in our analysis.

Another category of the proteins that require functional annotations are those from the Structural Genomics efforts. Quite often the functional aspects of the proteins that are experimentally solved under this initiative remain uncharacterized, in spite of the structure solution. There are around 333 *M.tb* structures deposited in the PDB that resulted out of structural genomics initiatives. Of these, 242 proteins are in the un-liganded form. In the NTPome, 3 proteins that belong to this category were identified using the default threshold of PMSmax ≥ 0.5. However, when partial similarities were considered through the PMSmin score, 5 more proteins were identified as hits. Table 2 shows the list of proteins in this category that were identified along with the PocketMatch scores. Alignments of the binding sites of *M.tb* proteins with the motifs for selected examples are shown in Supplementary Fig. S3. The geometry and the chemical nature of amino acids at the binding site clearly indicated the possibility of binding ATP in 5 proteins, GTP in 3 proteins, TTP in 2, and UDP in 2 proteins. This includes the possibility of the same protein binding to more than one NTP ligand, which is observed in nature for many proteins, including enzymes.

**Table 2:** List of proteins that belong to Structural Genomics initiatives that were identified in the NTPome. The binding site similarity scores of the individual proteins with their respective query NTP motif are also shown along with the ligand(s) matched.

Sl. no	RvID	Protein Name	PMSmax	PMSmin	Ligands
1	Rv0674	Conserved hypothetical protein	0.41	0.82	ATP, GTP
2	Rv0813c	Conserved protein	0.49	0.83	ATP
3	Rv1340	Probable ribonuclease RphA (RNase PH) (tRNA nucleotidyltransferase)	0.58	0.76	TTP
4	Rv1626	Probable two-component system transcriptional regulator regulatory proteins	0.51	0.89	ATP, GTP
5	Rv1825	Conserved protein	0.46	0.82	UDP
6	Rv1873	Conserved hypothetical protein	0.48	0.78	ATP
7	Rv2074	Possible pyridoxamine 5′-phosphate oxidase (PNP/PMP oxidase) (pyridoxinephosphate oxidase) (PNPOX) (pyridoxine 5′-phosphate oxidase)	0.52	0.79	TTP, UDP
8	Rv2717c	Conserved protein	0.41	0.79	GTP, ATP

### Identification of additional fold types for a possible NTP binding

A genome-wide survey for NTP binding sites carried out here enables an investigation of whether there are any additional folds that contain NTP binding sites. The fold space that is captured by experimentally solved structures for *M.tb* proteins binding to NTP ligands belongs to 23 different folds, such as TIM beta/alpha barrel, adenine nucleotide alpha hydrolase-like, nucleotide-diphospho-sugar transferases, P-loop containing nucleoside triphosphate hydrolases, and Protein kinase-like (PK-like), to name a few. The entire set of known NTP binding pockets from PDB were observed to belong to 145 different structural folds^35^. The set of 1,768 proteins in the *M.tb* NTPome belonged to a set of 350 different folds. A converse question to ask is- how many of these 350 folds were seen in PDB as NTP binding proteins from any species. In other words, how many of the 350 are in the set of 145 folds from the PDB dataset. 92 of the 350 folds were found to be in the PDB dataset as well, where as the rest 258 were newly identified fold-NTP binding site associations form this work. Some examples of these 92 folds are a) alpha-alpha superhelix fold - (representative in PDB: 1WA5), b) beta-Grasp fold - (representatives in PDB: 1Y8R and 1JWA), c) CO dehydrogenase flavoprotein C-domain like fold - (representative in PDB: 2CDU), d) Sigma2 domain of RNA polymerase sigma factors fold - (representative in PDB: 3LEV), and e) Dehydroquinate synthase-like fold -(representative from PDB: 1NVA). Thus, in addition to the 23 known folds for NTP binding in the *M.tb* NTPome which have experimentally solved structures, and 92 folds for which one or more fold templates are available in PDB for NTP binding, there are 258 new fold associations for NTP binding identified from this work. Some of the folds that fall in this category are listed in Table 3.

**Table 3:** 20 example folds in *M.tb* for fold-site associations that can be implicated for NTP binding.

Sl. No	Fold name	RvID	Protein name
1	6-phosphogluconate dehydrogenase C-terminal domain-like	Rv2573	Function unknown
2	Cytochrome P450	Rv3059/Rv0327c	Probable cytochrome P450/ Possible cytochrome P450 135A1 Cyp135A1
3	LolA-like prokaryotic lipoproteins and lipoprotein localization factors	Rv1270c	Lipoprotein LprA
4	Domain of alpha and beta subunits of F1 ATP synthase-like	Rv1293	Diaminopimelate decarboxylase LysA (DAP decarboxylase)
5	Amidase signature (AS) enzymes	Rv3375	Probable amidase AmiD (acylamidase) (acylase)
6	ClpP/crotonase	Rv2486	Probable enoyl-CoA hydratase EchA14 (enoyl hydrase)
7	Rhodanese/Cell cycle control phosphatase	Rv2291	Probable thiosulfate sulfurtransferase SseB
8	alpha/beta-Hydrolases	Rv1683	Possible bifunctional enzyme; long-chain acyl-CoA synthase and lipase.
9	Chelatase-like	Rv0265c	Probable periplasmic iron-transport lipoprotein
10	Profilin-like	Rv1354c	Conserved hypothetical protein
11	Pentein, beta/alpha-propeller	Rv2323c	Function unknown
12	Nitrite and sulphite reductase 4Fe-4S domain-like	Rv2391	Ferredoxin-dependent sulfite reductase SirA
13	Peptide deformylase	Rv0429c	Probable polypeptide deformylase Def (PDF)
14	FAH	Rv3536c	Probable hydratase
15	Thioesterase/thiol ester dehydrase-isomerase	Rv1532c	Conserved hypothetical protein
16	Zincin-like	Rv2367c	Conserved hypothetical protein
17	MFS general substrate transporter	Rv3331	Probable sugar-transport integral membrane protein SugI
18	Rhomboid-like	Rv1337	Probable integral membrane protein
19	MetI-like	Rv0929	Phosphate-transport integral membrane ABC transporter PstC2
20	Ketopantoate reductase PanE	Rv2573	Conserved hypothetical protein

## Discussion

In this work, we have sought to carry out a genome-wide survey so as to comprehensively identify a set of NTP binding proteins in *M.tb*. We predict that as many as 1,768 proteins coded in the *M.tb* genome would be capable of binding NTP and possibly constitute the NTPome, of which 72% are predicted to be ATP binding proteins. It was encouraging to see that most of the proteins that are bound to NTP ligands in the PDB database were correctly identified by our method, serving as positive controls.

Comparison of protein sequences forms an integral component of functional characterization of many proteins. There are a number of tools for sequence and whole-fold level comparison, which provides useful insights on the function of proteins^44–47^. Given these, a question that comes up is whether it is necessary to compare binding sites to identify ATP binding proteins in a given genome. The three-dimensional geometry and chemistry at the binding site of a protein generates its capability to recognize its cognate ligand, which is a prerequisite for all further functions in a number of proteins. The substructures at the functional sites in each protein, thus hold the key for its function. Although the sequence level classification relates the protein to a particular ancestry performing a particular function, at the level of binding sites, it could perform a completely different function^48,49^. In two of our recent studies involving a) all NTP binding proteins in PDB^35^, and b) sialic acid binding proteins^36^, we have found that a number of proteins that bind ATP share similarities in their binding sites, but do not share any similarity in their sequences or structural folds, clearly forming examples of convergent evolution. Another example, L-alanine dehydrogenase Ald (Rv2780), has no detectable sequence-level similarity to a known ATP-binding protein and hence is missed by sequence-based searches, but has a sub-structural motif that we could clearly link to ATP binding, and validate by DLAC as shown in Fig. 3. This protein has also been recently studied using X-ray crystallography and demonstrated to bind to ATP by us^50^ (PDB code:4LMP, to be published). To illustrate the importance of the surveying at the level of binding site structures, we asked how many of these would be identified by a sequence search alone. A search for NTP binding proteins in the tuberculist database results in identifying 410 proteins, simply based on the known annotations. Using sequence motifs such as the Walker motif as the query, again a sequence-based search in PROSITE^51^ for the motif results in identifying 161 proteins, of which many are of the same family. Put together, they identify only a portion of the NTPome.

Although other tools like NsitePred and ATPint that are available for nucleotide-binding prediction in proteins show comparable results for NTP predictions for some proteins like Rv1626, Rv0350, Rv3457c, and Rv3285, our method of site-based comparison and alignment served as a better tool to capture similarities for proteins like Rv0079, Rv1173, Rv2761c, and many others. Existing tools for identifying similarities at the binding sites like ProBis^52^ and SitesBase^53^ failed to capture the similarities for some of the top-ranking hits in our study, and also, for some proteins like Rv1379, and Rv1843c which are reported in literature to be NTP binding. Thus, the site-based approach as used in our study was not only able to capture similarities in these different example proteins, which are not detected by sequence-based methods, but also outperformed the existing sub-structural comparison tools in many cases.

In the recent years, chemical proteomics approaches have been developed which have identified a number of ATP binding proteins. Each method has its strengths and limitations based on the technology being used. Comparison of the lists of ATP binding proteins in *M.tb* detected by the chemical proteomics, activity-based probe, and desthiobiotin-ATP probe indicate that only a small fraction (14.5%) is common among them. This is clearly attributed to the detection range, limits and sensitivity. These limitations make it important not only to explore multiple experimental approaches but also computational approaches as an independent tool for genome-wide fishing. Computational methods based on binding site structures have the additional advantage of providing the list of residues, the exact location of the binding sites as well as an explanation of why the protein has such a capability. A limitation of the binding site-based methods however, is that they can detect NTP sites, only if a similar site is structurally characterized in some protein or the other. It is possible that some more structural motifs may be present in the proteins yet to be characterized. This in fact explains why some proteins identified by the chemical proteomics approaches are missed by our method. The number of these however is very small in comparison to the number of those that these methods have detected.

The functional insights provided by our study can be broadly classified into the following categories: (a) first clues for functional annotation, (b) adding high resolution detail to a known annotation, (c) detection of multiple binding sites in the same protein, indicative of allosteric regulation, and (d) suggesting ATP-motif based drug discovery for already known drug targets and other potential drug targets. The following examples illustrate these scenarios.

An important aspect of the annotation from our study is to provide first clues and assign functional associations to the proteins of unknown function, and proteins arising from the Structural Genomics consortium initiatives. In addition to the 4 hypothetical proteins that are experimentally shown to bind NTP ligands by us, an indirect evidence for Rv1626, for example, that this protein has a proposed annotation of phosphorylation-dependent transcriptional antitermination regulator lends vital support for our identification^54^.

Further, proteins like Rv0092, Rv1238, Rv1310, and Rv0342, to name a few, are identified to be NTP binding from our structure-based studies, for which, a sequence level of tuberculist annotation and experimental evidence exists for NTP binding by these proteins^9,55–59^. However, these proteins lack an experimentally solved structure, and hence, our study could provide major clues on the possible mode of NTP binding with the precise location of the binding with detailed residue information. Supplementary text A provides examples of few more proteins for which experimental evidence is available in literature. The prediction by us, in these cases, has not only led to the identification of an NTP-pocket in these proteins, but also, predicted the correct ligands for which an experimental support is available.

The function annotation in the NTPome can be potentially used for understanding structural basis of allostery in proteins, and their regulation by small-molecule ligands. Examples of function annotation in allosteric proteins are described in supplementary text B. Examples of proteins Rv1017c (prsA), Rv1098c (fum), Rv3676 (crp), Rv0998, Rv2996c (serA) and Rv3710 (leuA) are discussed with two possibilities of a) identifying a new additional location on the known allosteric protein, and b) suggesting a possible new allosteric modulator for the known allosteric protein.

A number of proteins in the NTPome are either known or are potential drug targets. For example, arabinosyltransferase (EmbC)^60^, ddla^21,22^, gyrA gyrB^61^, rpoB^62^, and ATP synthase^18^ all contain ATP binding sites. Identification of these sites and the type of motif in drug targets facilitate structure based lead discovery and lead optimization in cases where some candidate drugs are already known to bind at that site. In other cases, this may provide additional locations in the protein that may facilitate alternate target sites. From our previous work of identifying high-confidence drug targets in a multi-level, multi-scale target identification pipeline called targetTB^63^, 451 high-confidence drug target predictions were made, of which we note that 305 are in the current NTPome list.

In conclusion, we have identified 1,768 proteins in *M.tb*, which amounts to as much as 43% of the proteome, have the potential to bind nucleoside triphosphates. Using a sensitive sub-structural matching approach, we establish the superiority of site-based annotations over the sequence and whole fold based methods. We identify NTP binding in 294 proteins listed in the hypothetical and unknown function category. Using a chemical proteomics technique, we validate 47 proteins to be NTP binding, of which 4 are hypotheticals. As the rationale for understanding the binding of NTP in the different proteins is sub-structure based, we also provide useful insights on the binding mode of the ligand with the precise list of pocket locations. Our approach is generic and can be applied to studying other organisms as well, if structural models are available. To the best of our knowledge, this is a first study to report a genome-wide survey of NTP binding sites based on binding site sub-structures in *M.tb* or in any other species.

## Methods

### Data used in the study: NTP structural motifs and the *M.tb* pocketome

In a recent study in the laboratory, all known NTP binding sites from PDB, corresponding to 4,766 proteins (PDB deposition: as of May 2016) were analyzed, and grouped into 27 site-types through a structural bioinformatics approach, and site signatures or structural motifs were derived for each site-type^35^. The derived motifs were also determined to be specific towards NTP recognition. Each motif was used individually to query the *M.tb* pocketome, which is a dataset of 13,858 pockets, derived as previously described^64^. To obtain all the putative small-molecule binding pockets in *M.tb*, a structural modeling of the *M.tb* proteome was required at the first step which was carried out in a previous study. From this study, structural models for 2,877 proteins including 324 crystal structures and 2,737 comparative models were available which accounted for 70% of the total proteome. The model building exercise for proteins was carried out in such a way that it is independent of whether a protein has an experimentally solved structure already deposited in the PDB or not. For those proteins which already had experimentally solved structures in PDB, it was compared whether our generated model was able to reproduce the structural parameters of the solved structure. This step resulted in obtaining all the structural models accurately for the proteins that were already deposited in PDB with exact protein length, minimal RMSD and favorable stereochemical properties. This served as the first level of confidence and accuracy of the generated models. For all the other models generated for which an experimentally solved structure was not available, it was checked that they fulfill all the structure verification methods including secondary structure compatibility, statistical potential and other stereochemical properties. Thus, with these high-confidence structural models in first hand, we generated the *M.tb* pocketome. For this, all putative small-molecule binding pockets were identified based on a consensus of three different algorithms; PocketDepth^30^ (geometry-based), LigSite^65^ (evolutionary-based), and SiteHound^66^ (energy-based). Thus, a pocket was considered only if it seen identified by all the three different methods, which led to an overall of 13,858 high-confidence pockets^25,64^, which was used as the *M.tb* pocketome for searching for the NTPome.

### Binding site comparison and alignments

The binding sites were compared in an all-vs-all manner by using an in-house algorithm for binding site similarity called PocketMatch^32^. The algorithm captures chemistry and geometrical shape of the binding site. Each binding site is represented by 90 lists of sorted distances, and subsequently aligned incrementally to obtain a similarity score. This score is called the PocketMatch score (PMS), which is scaled between 0 and 1, where 1 indicates identity. We have previously shown that, a score of PMSmax > 0.4 indicates biologically meaningful similarities, since it implies significant similarity in the whole site^31,34^. An added advantage of using PocketMatch is that it also reports a local score called the PMSmin score which reflects a local sub-structural match, when part of the site in the hit matches with a part or the whole site in the query, which is also utilized as a combination along with PMSmax in this study. ATP binding sites are large in some crystallographically determined ATP sites which have about 25 to 30 residues in them, while some other proteins in the same superfamily contain only 12 to 18 residues. It is thus important to consider partial similarities. PMSmin indeed served this purpose and identified cases where there was a significant similarity in a portion of the pocket, typically encountered in sites of dissimilar sizes. A high PMSmax score implied similarly sized pockets containing similarly positioned residues of similar chemical properties, while high PMSmin scores indicate significant similarity in part of the sites. This happens often when the sizes of the two pockets in the pair being studied are unequal. Thus, a combination of PMSmax and PMSmin serves as a useful scheme to identify similarities in such cases. We chose a PMSmax cutoff of 0.5 and a minimum number of 5 matched residues, to consider two binding sites as similar. The significance of using PMSmin scores for identifying similar NTP binding sites is shown in section 2.5.

Binding site alignments at the structural level were performed using another in-house algorithm called PocketAlign^33^. Pymol (version 1.2r1 from www.pymol.org) was used for structural analyses and generating images. Sequence-based ATP site prediction tools NsitePred^67^ and ATPint^68^ were used for comparing with our site-based NTP predictions. Tuberculist^69^ database was used for comparing the annotations, and KEGG mapper^70–72^ was used for highlighting the pathway enrichment.

### Dye-ligand affinity chromatography

Experimental testing of ATP binding of *M.tb* proteins was carried out using a DLAC protocol, as described earlier by one of our laboratories (Kim *et al*.^40^, Kim *et al*.^41^ and Roberts *et al*.^50^). Briefly, this involves elution of native proteins of *M.tb* cell extract based on ligand-affinity chromatography followed by 2D-gel electrophoresis and mass-spectrometric characterization. An independent nucleotide interaction analysis using expressed and purified proteins was also carried out in selected cases. Proteins from a crude cytosolic extract were bound to a resin matrix and selectively eluted out using nucleotide ligands. Independently, specific protein-ligand interactions for selected proteins were examined using purified recombinant proteins. A detailed description of the methods is provided in Supplementary material as supplementary methods.

In brief, 100 mg of crude cytosolic extract of *M.tb* H37Rv was adsorbed to a 10 ml Cibacron F3GA Blue affinity column. A column buffer (CB) containing 50 mM KH_2_PO_4_ pH 7.5, 1 mM MgCl_2_ and 2 mM DTT was used to wash the column extensively to remove the low-affinity and unbound proteins before eluting with ligands. It was seen that approximately 40% of the whole cytosolic extract was bound to the resin, which was determined by a standard Bradford assay. The solubilized protein was recovered, and a ligand-specific elution, which was applied in series, was carried out using 5 ml of each of the ligands with a wash with CB between each elution. Peak ligand fractions were pooled and the proteins were precipitated by adding 100% iced trichloro acetic acid (TCA). Precipitated proteins were recovered by centrifugation and the recovered proteins were fractionated by 2D-SDS-PAGE and mass spectrometry. The specificity of ligand interactions was further examined by testing the elution of purified recombinant proteins from the dye-resin by each of the individual ligands tested.

### Data availability statement

Data and methods used in this study can be accessed freely from their original databases described in the methods section.

## Electronic supplementary material

Supplementary Table ST1

Supplementary material- methods, figures and table ST2

Supplementary Table ST3
