# Publisher Correction: A genome-wide structure-based survey of nucleotide binding proteins in *M. tuberculosis*

**DOI:** 10.1038/s41598-020-77427-x

**Published:** 2020-11-19

**Authors:** Raghu Bhagavat, Heung-Bok Kim, Chang-Yub Kim, Thomas C. Terwilliger, Dolly Mehta, Narayanaswamy Srinivasan, Nagasuma Chandra

**Affiliations:** 1 Department of Biochemistry, National Mathematics Initiative, Bangalore, India; 2 Molecular Biophysics Unit, Indian Institute of Science, Bangalore, 560012 India; 3 Bioscience Division, Los Alamos National Laboratory, Los Alamos, New Mexico 87545 USA

Correction to: *Scientific Reports* 10.1038/s41598-017-12471-8, published online 02 October 2017

Two of the supplementary files containing Supplementary Tables ST1 and ST3 were omitted from the original version of this Article. This has been corrected in the HTML version of the Article; the PDF version was correct at time of publication.

