# Supplementary material for: A genome-wide structure-based survey of nucleotide binding proteins in M. tuberculosis
Source: Sci Rep. 2017 Oct 2;7:12489. doi: 10.1038/s41598-017-12471-8 (PMC5624866; doi:10.1038/s41598-017-12471-8)
Supplement: Supplementary file 2 — Supplementary material- methods, figures and table ST2 [file 41598_2017_12471_MOESM2_ESM.pdf]

Supplementary information for 'A genome-wide structure-based survey of nucleotide binding proteins in *M. tuberculosis*'

Raghu Bhagavat[1], Heung-Bok Kim[2], Chang-Yub Kim[2], Thomas C. Terwilliger[2], Dolly Mehta[1], Narayanaswamy Srinivasan[3], and Nagasuma Chandra[1]*

[1]Department of Biochemistry, National Mathematics Initiative, [3]Molecular Biophysics Unit Indian Institute of Science, Bangalore -560012, India
Tel: +91-80-22932892; Fax: +91-80-23600814

[2]Bioscience Division, Los Alamos National Laboratory, Los Alamos, New Mexico 87545, U.S.A.

**E-mail: nchandra@biochem.iisc.ernet.in*

Supplementary methods

Dye-ligand affinity chromatography

A) Ligand-specific elution of native proteins of *M.tb* cell extract from Cibacron Blue F3GA resin

A crude cytosolic extract, 100 mg (BEI resources, NIAID, NIH: NR-14834) from *M.tb* strain H37Rv was adsorbed to a 10 ml "Affigel" Blue Gel (Cibacron F3GA Blue) affinity column (BioRad). The affinity column was washed extensively with column buffer (CB; 50 mM KH_2PO_4, pH 7.5, 1 mM $MgCl_2$ and 2 mM DTT or CB with additional 0.2 % ω-Undecylenyl-ß-D-maltopyranoside (ωUDM) for membrane fraction) to remove unbound and low-affinity proteins prior to ligand elution. Approximately 40% of total cytosolic protein bound to the resin, as determined by Bradford assay. An aliquot of the resin-bound protein was extracted for

subsequent 2D-gel analysis prior to elution; ~100 mg resin was extracted in 250 µl urea sample buffer (USB; 8% urea, 2% NP-40, 18 mM DTT), and the solubilized protein recovered in a spin column (Costar, Fisher Scientific). Ligand-specific elution was carried out using 5 ml of each ligand at 1 mM in CB. Ligands were applied in series, and the column was washed with 20 ml CB between elutions with ligands. Up to 20 different ligands were used to elute proteins from a single column. During elution, column was monitored using an in-line flow cell set at 260nm. Peak ligand fractions were pooled and the protein precipitated by addition of 100% iced TCA to a final concentration of 20%. Precipitated proteins were recovered by centrifugation, washed with acetone, and solubilized in 300 µl urea sample buffer. Recovered proteins were fractionated by 2-dimensional IEF-SDS-PAGE, using 13 cm pH 3-10 NL Immobilon gradient strips in the first dimension (Pharmacia Biotech IPGphor system), and 15% SDS slab gels in the second dimension. Proteins on the gel were stained with Coomassie Brilliant Blue R250, and protein spots were excised for tryptic digestion using a modified procedure of Chang *et al.* (Chang *et al.*, 2000). The excised gel spots were stored in 100 µl of HPLC-grade water at 4°C until subsequent process. The spots were then minced and washed with 25 mM NH_4HCO_3 in 50% (v/v) acetonitrile. The gel pieces were allowed to dry and then rehydrated in 25 mM NH_4HCO_3 with 0.5 to 1.0 µg of trypsin at 37°C overnight. After digestion, the digestion solution was separated from the gel slices, and the gel slices were washed with HPLC-grade water once and with 50% (v/v) acetonitrile, 5% (v/v) trifluoroacetic acid three times at room temperature to extract the peptides further. Pooled extracts (including the digestion solution and both the aqueous and organic washes) were concentrated using a Speed-Vac for mass spectroscopy analysis.

A membrane fraction was isolated from *M.tb* H37Rv whole cell lysate (BEI resources, NIAID, NIH: NR-14822); 2 ml of breaking buffer (PBS pH7.4, 1mM EDTA, Pepstatin, Leupeptin &

PMSF) to 100 mg whole cell lysate and centrifuged at 27,000 g for 20 min. The supernatant was transferred to a new tube and centrifuged 3 more times. The final supernatant was transferred to a new tube and re-centrifuged at 100,000 g for 4 hours. The supernatant was removed and the pellet was washed with 1ml CB. The washed pellet was re-suspended with 1 ml of CB. To solubilize this membrane fraction for DLAC analysis, 1 ml of 12 mM ω-Undecylenyl-ß-D-maltopyranoside (ωUDM) in CB was added to 1 ml membrane fraction and vortexed at 4°C for 1 hour. The soluble membrane fraction was prepared by collecting the supernatant after centrifugation at 200,000 g for 2 hours. The DLAC experiment was performed by adding 100 µl of Cibacron F3GA Blue resin slurry (1:1 volume ratio with resin and CB) to a spin column and wash with 400 µl of 12 mM ωUDM in CB (ωUDM-CB) by spin at 14,000 g for 1 min. The membrane proteins were adsorbed on to the resin by adding 100 µl membrane fraction and gentle vortexing at 4°C for 1 hour. The unbound proteins were collected by spin at 14,000 g for 1 min, and washed with 400 µl of ωUDM-CB by four cycles of brief vortex and spin at 14,000 g for 1 min. The NTP interacting proteins were eluted by adding 100 µl of 5 mM each NTP ligand per column and vortexing at 4°C for 1 hour followed by spin at 14,000 g for 1 min. The protein concentration of each collected tube was measured by Bradford assay. The 30 µg of protein fraction was added in a siliconized tube, and 5 µl of 5 M urea and newly prepared 5 µl of 200 mM DTT were added to vortex and micro-centrifuge briefly. After incubation of the tube at 56°C for 1 hour, 20 µl of fresh Iodoacetamide was added and incubated at room temperature for 30 min in dark. Fresh trypsin prepared by mixing 100 µl of 50 mM acetic acid to 100 µg of lyophilized 1 µg/µl trypsin was added to the tube to make 1:10 mass ratio of trypsin:protein, and incubated at 37°C for 16 hours for mass spectroscopy analysis. Tryptic peptide masses were measured by analyzing one-twentieth of each concentrated sample after digestion using a matrix-

assisted laser desorption-ionization delayed extraction reflectron time-of-flight (MALDI-DE-TOF) mass spectrometer equipped with a nitrogen laser (set at 337 nm) (Voyager-DE STR, PE Biosystems, Framingham, MA). Peptides were co-crystallized 1:1 (v/v) with matrices consisting of saturated α-cyano-4-hydroxycinnamic acid prepared in 50% (v/v) acetonitrile/1% (v/v) trifluoroacetic acid. All MALDI spectra were either externally calibrated using a standard peptide mixture or internally calibrated using trypsin auto-proteolysis products. Mono-isotopic masses from all spectra recorded for a given peptide are used for matching. For several peptides that exhibited the highest pseudo-molecular ion abundance on MALDI mass spectra, partial amino acid sequence was determined using post-source decay analysis.

Matching of experimental results (measured peptide mass values) with theoretical digests and sequence information obtained from various databases was performed using two sequence database search programs, MS-Fit and MS-Tag (http://prospector.ucsf.edu/prospector/ mshome.htm). MS-Fit allows the user to match the observed tryptic peptide masses of an unknown protein to the expected peptide masses of any protein for which amino acid or nucleotide sequence information is available. Database queries were carried out for mono-isotopic peptide masses using the following parameters: peptide mass tolerance of ±50 ppm, equivalent to 0.1 Da for a 2-kDa peptide; the maximum number of missed tryptic cleavages of 2 or 3; and modifications including conversion of peptide N-terminal Gln to pyro-Gln, oxidation of Met, acetylation of the N terminus, and modification of Cys by acrylamide.

Database searches using MS-Tag to match post-source decay (PSD) fragment ions (along with the mass of a precursor ion) used the following parameters: precursor ion mass tolerance of ±100 ppm (measured by MALDI-MS) and PSD fragment ion mass tolerance of ±1,500 ppm.

Databases searched included protein databases such as the non-redundant NCBInr compiled by the National Center for Biotechnology Information.

B) Nucleotide interaction analysis by DLAC using expressed and purified proteins

The *M.tb* cell extract proteins identified for interaction with nucleotides were amplified by PCR from *M.tb* H37Rv genomic DNA for each corresponding gene, using Pfu polymerase (Stratagene), and the following primers: 5'-TACTTCCAATCCAATGCGAT G+N-terminal 20 nucleic acids coding region of target protein-3' (forward) and 5'-TTATCCACTTCC AATGTTA+C-terminal 20 nucleic acids coding region of target protein-3' (reverse). The underlined bases were to generate ligation-independent cloning (LIC) sites for the pMCSG7 vector. The pMCSG7 vector was digested with the SspI restriction enzyme (Promega), and the amplified and purified PCR product and a singly digested pMCSG7 vector were treated with T4 DNA polymerase (Novagen). The 50 µl of *E. coli* NovaBlue cells (Novagen) was transformed with the self-annealed PCR product and pMCSG7 vector. The insert of genes in plasmids was confirmed by DNA sequencing. *E. coli* BL21 (DE3) cells were used to express the cloned genes. Cells were grown at 37°C in LB medium (Sigma) containing 100 µg/ml ampicillin, induced with 1 mM IPTG when OD600 reached 1.0, and grown at 25°C overnight in a shaking incubator set at 250 rpm. The cells were harvested and stored at -80°C. The expression of each protein was checked by SDS-PAGE. For purification of expressed proteins, frozen cells were thawed on ice and resuspended in lysis buffer (20 mM Tris-HCl pH 8.0, 200 mM NaCl, 1 mM PMSF, 1 mg/ml DNase, 1 mM $MgCl_2$). Lysates were sonicated and then centrifuged with 3,000 g at 4°C for 30 min. The supernatant was filtered through a 0.45 µm pore membrane (stericup, Millipore) and loaded on a 5 ml Ni-NTA superflow affinity column (Qiagen). After being washed with buffer A (20 mM Tris-HCl pH 8.0, 200 mM NaCl), the target protein was eluted by buffer B (buffer A

plus 500 mM imidazole). To remove the contaminants, eluted fractions were further purified on a Superdex-75 gel filtration column (GE Healthcare Inc.) using buffer C (10 mM Tris-HCl pH 8.0, 150 mM NaCl, and 1 mM DTT). A centrifugal concentrator (Millipore) was used to concentrate the pooled protein fractions to 5–15 mg/ml, as measured by Bradford reagent (Bio-Rad). Protein purity was confirmed by SDS-PAGE and densitometry.

To evaluate recombinant proteins for their specific ligand-binding properties, we followed a modified version of the protocol described in Kim *et al.* (Kim et al., 2009). Briefly, individual purified proteins were diluted to 1–2 mg/ml in column buffer (CB), and 100 μg protein was adsorbed to multiple 50 μl aliquots of F3GA resin (BIO-RAD) in 2 ml spin-columns. Aliquots (F3GA resin + proteins) were vortexed at 4°C for 1 hour for binding, followed by recovery of unbound protein (flow-through fraction) and washing of the resin five times with 500 μl CB. Spin-columns were centrifuged for 30 s at 10,000 g to recover fractions and change solutions. Individual spin-columns containing resin-bound proteins were then incubated (as for protein binding) with 50 μl, 1 mM ligand in CB, and the eluate fraction was recovered by centrifugation. Aliquots of initial protein, flow-through, and eluate fractions were diluted with 1:1 volume ratio with 2x SDS sample buffer, and 15 μl was loaded in equal proportion (equivalent to 1 μg input protein) on 10% SDS-PAGE to confirm the proteins eluted by interaction with each nucleotide ligands.

Supplementary text A (quoted in Discussion in the main manuscript)

It was interesting to see that for proteins Rv1023, Rv1065 and Rv1843c, which were identified in the top-ranking list in our study, have also been experimentally tested for binding ATP in the first two proteins and GMP for the third (Wolfe, L. M. *et al.,* 2013, Kanehisa, M. *et al*., 2014 and Roberts *et al.,* 2008). This also shows that our method is not biased towards predicting a particular ligand, as, for Rv1023 and Rv1065, an ATP and GMP ligand was correctly predicted, and that our method could capture the subtleties of discriminating the different nucleotides, wherever possible. Also, for proteins, Rv1379 (probable pyrimidine operon regulatory protein, PDB code 1W30), and Rv1626 (probable two-component system transcriptional regulator, PDB code 1S8N), it was seen that both have experimentally solved structures in the apo-form, and had no information about the ligand binding. Again, in the above two cases, the possible mode of NTP binding and the set of pocket residues is deciphered from our analysis. While a uracil-phosphoribosyltransferase activity was reported for Rv1379 under the GO-classification, there was no direct information of a possible NTP binding for Rv1626, which Rupp and co-workers (Kantardjieff *et al.,* 2005) and Tucker and co-workers (Tyagi and Sharma, 2004; Morth *et al*., 2004) have shown experimentally that Rv1379 and Rv1626 indeed binds UMP and ATP respectively. Here also, the correct nucleotide-ligands were predicted by our method.

Supplementary text B (quoted in Discussion in the main manuscript)

For protein Rv1017c, annotated as a probable ribose-phosphate pyrophosphokinase, there is no experimentally solved structure available. While our structural model and the putative NTP binding site residues information provides the first level of useful information for understanding the structural basis of ATP binding in this protein, an additional pocket location on the same

subunit suggests a potential allosteric site in the same protein. It is also interesting to note that the two distally located pockets show ability to bind ATP and UDP respectively. For protein Rv1098c which exhibits allostery (Kasbekar *et al.*, 2016), two distally located pockets which have potential to bind ATP and UDP respectively are identified in the NTPome. Rv3676 (crp) has an experimentally solved structure bound to the activator, cyclic-AMP. From our analysis, we identify an additional site on Rv3676 which shows ATP binding potential, suggesting a possible allosteric modulation by ATP in this additional site. In case of Rv0998, the experimentally solved structure is bound to CoA and cyclic-AMP ligands. We identify that the CoA binding site in Rv0998 shares significant similarity to bind ATP, thus suggesting a possible allosteric modulation by ATP at the CoA site. The arginine repressor argR protein is allosterically regulated by arginine (Strawn, R. *et al.,* 2010) which acts as an activator. In our NTPome, it is interesting to see that this protein shows potential for ATP binding at an additional site which is not the activator arginine binding pocket. Even in case of Rv2996c (serA), which is allosterically inhibited by serine (Asención Diez *et al.,* 2015), we identify two additional pockets on this protein for a possible ATP and UDP binding at different locations, suggesting a possible modulation by ATP and UDP. We also suggest a possible GTP and ATP binding at different locations for protein Rv3710 (leuA), which exhibits allostery (Koon *et al.,* 2004).

Chang, W.W., Huang, L., Shen, M., Webster, C., Burlingame, A.L., Roberts, J.M.. Patterns of protein synthesis and tolerance of anoxia in root tips of maize seedlings acclimated to a low-oxygen environment, and identification of proteins by mass spectrometry. *Plant physiology* **122**, 295-318 (2000).

Kim, C.-Y. *et al.* Analysis of nucleoside-binding proteins by ligand-specific elution from dye resin: application to Mycobacterium tuberculosis aldehyde dehydrogenases. *J. Struct. Funct. Genomics* **10,** 291–301 (2009).

Wolfe, L. M. *et al.* A Chemical Proteomics Approach to Profiling the ATP-binding Proteome of Mycobacterium tuberculosis. *Mol. Cell. Proteomics MCP* **12,** 1644–1660 (2013).

Kanehisa, M. *et al.* Data, information, knowledge and principle: back to metabolism in KEGG. *Nucleic Acids Res.* **42,** D199–D205 (2014).

Roberts, J., Cecilia, W., Terwilliger, T. & Kim, C.-Y. High-throughput Analysis of Nucleoside and Nucleotide-binding by Proteins. *Systems Chemistry* (2008).

Kantardjieff, K. A. *et al.* Structure of pyrR (Rv1379) from Mycobacterium tuberculosis: a persistence gene and protein drug target. *Acta Crystallogr. D Biol. Crystallogr.* **61,** 355–364 (2005).

Jaya Sivaswami Tyagi and Deepak Sharma. Signal transduction systems of mycobacteria with special reference to M. tuberculosis. *Current Science* **86,** (2004).

Morth, J. P., Feng, V., Perry, L. J., Svergun, D. I. & Tucker, P. A. The crystal and solution structure of a putative transcriptional antiterminator from Mycobacterium tuberculosis. *Struct. Lond. Engl. 1993* **12,** 1595–1605 (2004).

Kasbekar, M. *et al.* Selective small molecule inhibitor of the Mycobacterium tuberculosis fumarate hydratase reveals an allosteric regulatory site. *Proc. Natl. Acad. Sci. U. S. A.* **113,** 7503–7508 (2016).

Strawn, R. *et al.* Symmetric Allosteric Mechanism of Hexameric Escherichia coli Arginine Repressor Exploits Competition between L-Arginine Ligands and Resident Arginine Residues. *PLOS Comput. Biol.* **6,** e1000801 (2010).

Asención Diez, M. D. *et al.* Allosteric regulation of the partitioning of glucose-1-phosphate between glycogen and trehalose biosynthesis in Mycobacterium tuberculosis. *Biochim. Biophys. Acta BBA - Gen. Subj.* **1850,** 13–21 (2015).

Koon, N., Squire, C. J. & Baker, E. N. Crystal structure of LeuA from Mycobacterium tuberculosis, a key enzyme in leucine biosynthesis. *Proc. Natl. Acad. Sci. U. S. A.* **101,** 8295–8300 (2004).

Supplementary figures

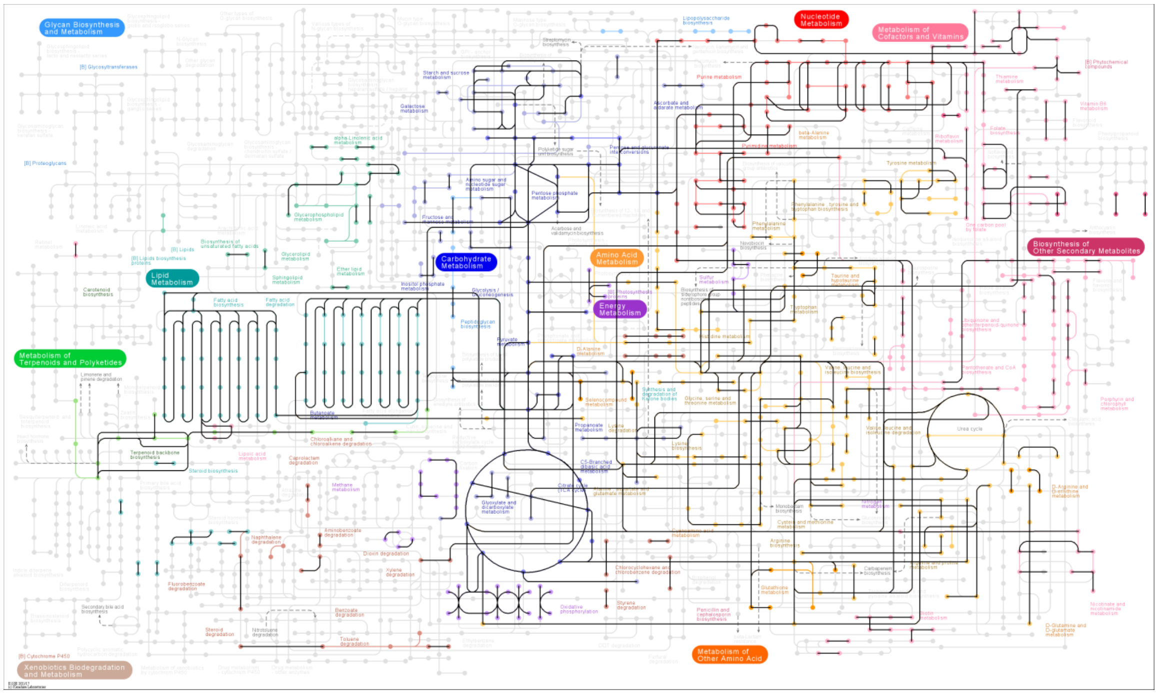

Supplementary figure S1: KEGG mapper showing the various pathways that are enriched in the NTPome. It can be seen that lipid metabolism, nucleotide metabolism, amino acid metabolism and carbohydrate metabolism form the major pathways that are more represented in the NTPome.

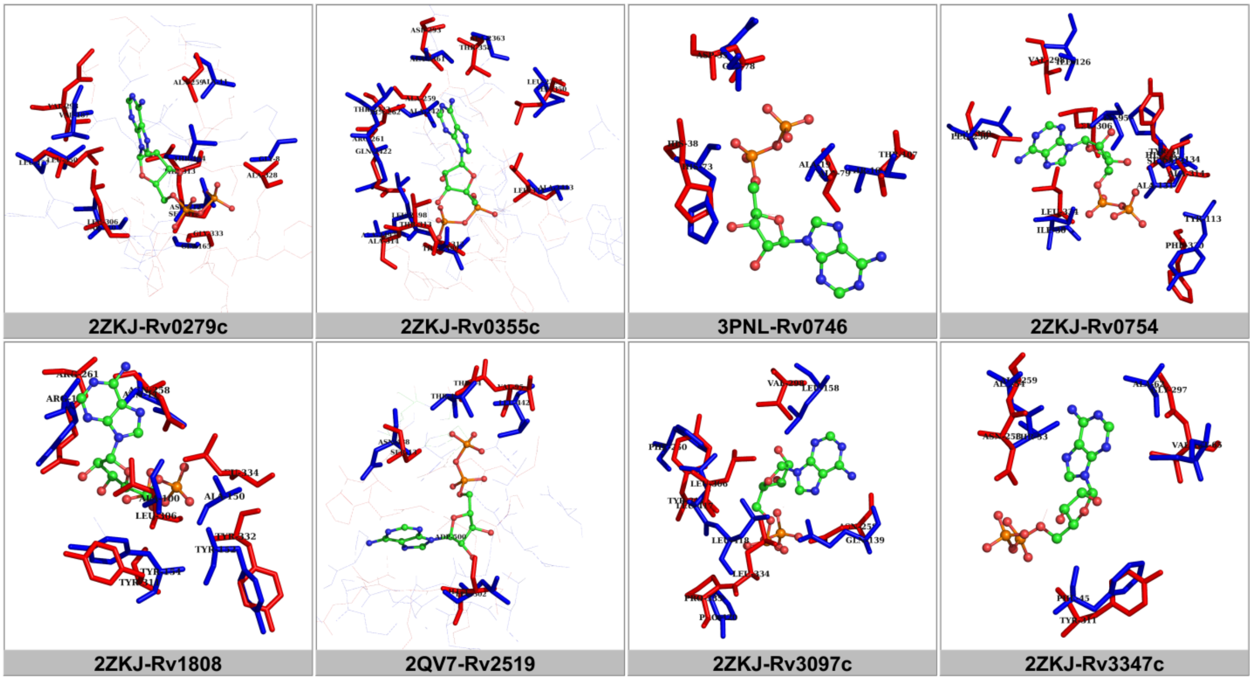

Supplementary figure S2: Pair-wise binding site alignments for 8 Mtb proteins belonging to PE/PPE family of proteins shown in blue, with their respective NTP motif sites in red stick representations, and the ligand in ball and stick representation.

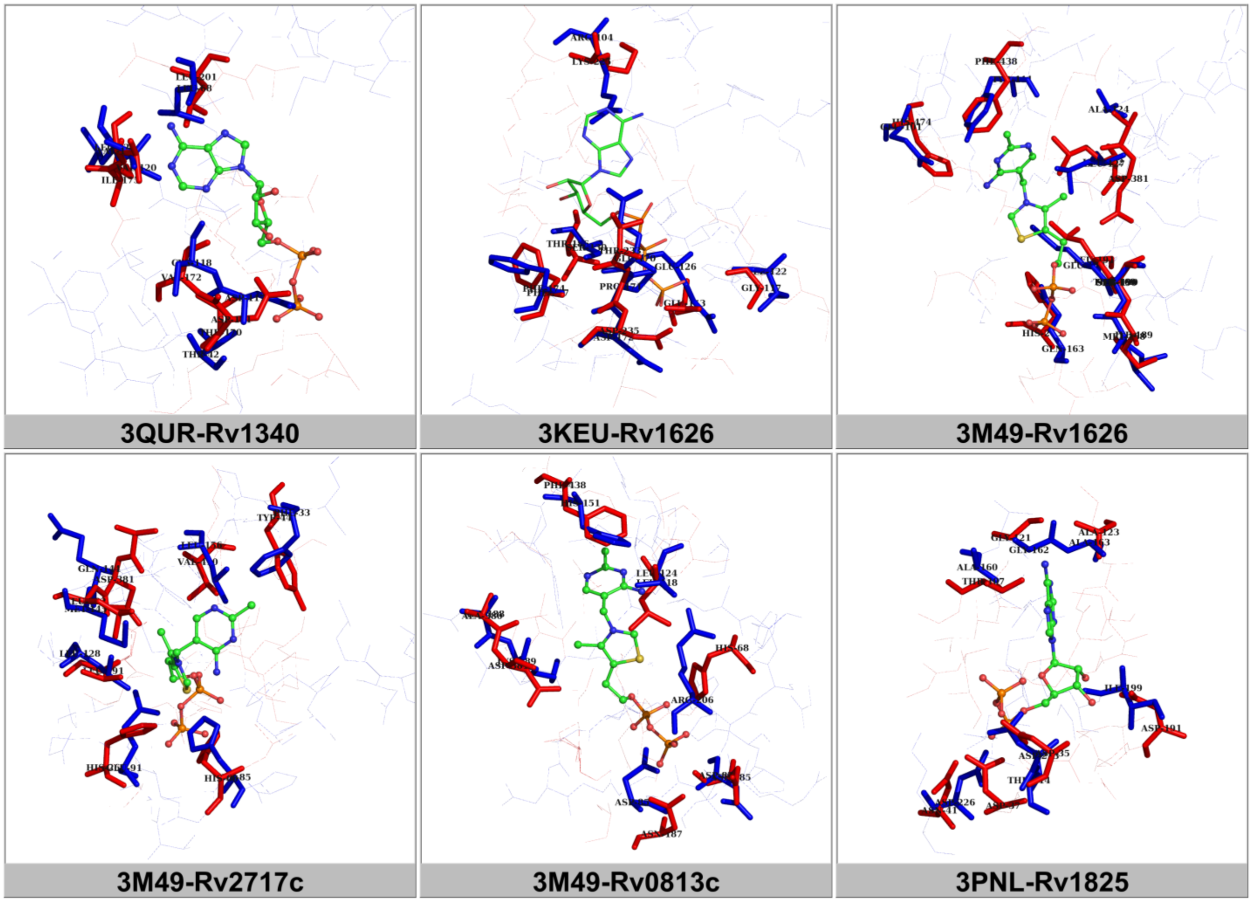

Supplementary figure S3: Pair-wise superpositions of the NTP motifs with five Mtb proteins belonging to Structural Genomics consortium are shown in red and blue stick representations respectively. The RvIDs for each of the proteins is mentioned in the panel with the ligand shown in ball and stick representation in all panels. The NTP motif used as reference is labeled by the representative from one of the site-types that was identified from a previous study. It can be seen that Rv1626 shared similarities with two different site-types 3KEU and 3M49, and hence both are shown.

Supplementary tables

ST1 and ST3 are excel files

Supplementary table ST1 lists all the 1768 proteins in the NTPome, along with their functional categories.

Supplementary table ST1 (sheet 2) lists the proteins under the category of hypotheticals and unknown function that were identified as hits for NTP binding.

Supplementary table ST3 showing the highest PMS score each of the 1,768 proteins have with the queried NTP motif. It is possible that a protein shares similarity with more than one NTP motif, but for clarity that with the highest similarity score is listed.

Supplementary table ST2: List of proteins identified as hits for NTP binding at different thresholds of the PocketMatch similarity scores. The list of 2183 proteins identified at a combined threshold of PMSmax ≥0.4 + PMSMin ≥0.6 could be useful as well, considering the high PMSmin. But, we chose the optimum threshold of PMSmax ≥0.5 to reduce the number of false positives, and hence constitute the number of proteins in NTPome.

Sl. No	PMS thresholds	# of protein hits
**1**	**PMSmax ≥0.5**	**1768**
2	PMSmax ≥0.4 + PMSMin ≥0.6	2183
3	PMSmax ≥0.4 + PMSmin ≥0.75	237
4	PMSmax ≥0.6	136
